# Owned and Unowned Dog Population Estimation, Dog Management and Dog Bites to Inform Rabies Prevention and Response on Lombok Island, Indonesia

**DOI:** 10.1371/journal.pone.0124092

**Published:** 2015-05-01

**Authors:** Ana Mustiana, Jenny-Ann Toribio, Muktasam Abdurrahman, I. Wayan Suadnya, Marta Hernandez-Jover, Anak Agung Gde Putra, Michael P. Ward

**Affiliations:** 1 The University of Sydney, Faculty of Veterinary Science, Camden, NSW, Australia; 2 Research Center for Rural Development, Faculty of Agriculture, University of Mataram, Lombok, Indonesia; 3 Graham Centre for Agricultural Innovation (NSW Department of Primary Industries and Charles Sturt University), School of Animal and Veterinary Sciences, Wagga Wagga, NSW, Australia; 4 Disease Investigation Centre Denpasar, Ministry of Agriculture, Denpasar, Indonesia; University of Missouri, UNITED STATES

## Abstract

Although Indonesia has been rabies-infected since at least the 1880s, some islands remain rabies-free, such as Lombok. However, due to its adjacency to rabies-infected islands such as Bali and Flores, there is considerable risk of a rabies incursion. As part of a rabies risk assessment project, surveys were conducted to estimate the size of the dog population and to describe dog management practices of households belonging to different ethnic groups. A photographic-recapture method was employed and the number of unowned dogs was estimated. A total of 400 dog owning households were interviewed, 300 at an urban site and 100 at a rural site. The majority of the interviewed households belonged to the Balinese ethnic group. Owned dogs were more likely male, and non-pedigree or local breed. These households kept their dogs either fully restricted, semi-free roaming or free-roaming but full restriction was reported only at the urban site. Dog bite cases were reported to be higher at the urban site, and commonly affected children/young adults to 20 years old and males. A higher number of unowned dogs was observed at the urban site than at the rural site. Data generated within these surveys can inform rabies risk assessment models to quantify the probability of rabies being released into Lombok and resulting in the infection of the local dog population. The information gained is critical for efforts to educate dog owners about rabies, as a component of preparedness to prevent the establishment of rabies should an incursion occur.

## Introduction

Dogs are closely associated with humans, being kept by households as companions and are often considered as part of the family, especially in developed countries [1,2]. In some communities, for instance Indigenous communities in Australia and some ethnic groups in Indonesia, dogs play an important role in the culture of the community [3,4]. However, when dogs are not fully provided with food and shelter, then they will roam if not confined. Semi-roaming and free-roaming dog populations can create risks for public health due to zoonotic diseases, such as rabies [5]. Thus, understanding the dog–human relationship in a community is critical for planning rabies prevention and control.

Rabies was first reported in Indonesia during the 1880s [6], and is still maintained in the domestic dog populations of the majority of Indonesian islands today. The number of human rabies deaths in Indonesia during 2012 was reported to be 662 [7]; however, this is considered an underestimation of the national human loss due to unreported cases (Syafrison Idris *pers*. *comm*., 2013). Rabies eradication efforts in most infected provinces have not been successful for several reasons, but most importantly the difficulties encountered in vaccinating free-roaming dogs [8–10]. The failure of rabies control has led to further spread of the disease to previously rabies-free islands; recently this includes Bali (2008), Nias and Larat Islands (2010) and Kisar Island (2012) [7]. With the absence of wildlife rabies reservoirs in Indonesia [11], the spread of rabies from island to island across the archipelago occurs via human-mediated movement of dogs incubating rabies [12].

Consideration of the role of the dog, level of dog ownership and nature of dog management in a community appears warranted when planning for rabies control and prevention in Indonesia. Rabies was successfully eradicated from areas of Java, where the control program was aided by a high proportion of confined owned dogs compared to free-roaming dogs that allowed vaccination of a sufficient proportion of the dog population to prevent rabies transmission [13]. Community attitudes of the majority Muslim Javanese communities towards dogs − including lower dog ownership and less tolerance of free-roaming dogs − assisted the control effort. In contrast, community culture may be an obstacle to rabies control activities in areas where dogs are highly valued. For example, dogs are used for sport (pig hunting) in some communities and many hunters believe that vaccination could affect their dogs’ performance, thus they reject dog vaccination [10]. Dog population reduction through culling of free-roaming dogs, when implemented in communities on Flores and Bali islands, led to people moving their dogs out of the community to protect them and to acquiring new puppies and adopting free-roaming dogs from other areas. Thus on both these more recently infected islands, dog culling following rabies introduction − with 295,565 dogs (48% of the island’s dog population) killed from 1998–2001 on Flores [14], and 108,000 dogs from 2008–2011 on Bali [8] − did not produce a reduction in rabies cases or prevent rabies spread across the island. On Bali, a decrease in rabies cases was not observed until the implementation of systematic island-wide vaccination of dogs [8]. To be effective such a dog vaccination campaign needs to achieve the recommended 70% vaccination coverage [15], and implementation requires knowledge of the dog population in terms of number and structure (to plan and ensure an adequate and timely vaccine supply), ability to capture roaming dogs and to identify vaccinated dogs, education campaigns for dog owners and for communities, good quality vaccines, media support and effective general coordination of the vaccination campaign [16,17,18]. Internationally, the control and prevention of urban rabies has been based on dog vaccination and population management [19]. Both vaccination and population control programs require data on the distribution and numbers of free-roaming dogs including understanding of differences between rural and urban environments; thus, estimation of the free-roaming dog population size is a necessary first step in planning rabies control programs [17,18,20,21].

Lombok Island, a rabies free island in eastern Indonesia situated between rabies infected Bali Island (35km east) and Flores Island (300km west), is at risk of rabies introduction. Understanding the presence of dogs within ethnic groups living on Lombok Island, and management of their dogs, is necessary to develop strategies to prevent and to control a potential rabies incursion. However, no study in Indonesia has yet been conducted on rabies-free islands to understand the role of ethnicity. Our overall program goal was to investigate pathways for the entry of rabies to Lombok via dog movement, and assess the probability of rabies entry and exposure to the susceptible dog population on Lombok. To inform this risk assessment, a survey of dog-owning households and photographic capture methods were conducted. Our objectives were to describe dog management practices of dog-owning households belonging to different ethnic groups at an urban and a rural site on Lombok Island, and to describe dog bite occurrence and estimate dog population size at these sites.

## Methods

### Study sites

Lombok is one of two larger islands within West Nusa Tenggara (NTB) province, located in the eastern part of Indonesia. It has a total land area of about 4,725 km^2^ and is inhabited by 3,200,686 people, with fishing, agriculture and tourism the important sources of income [22]. Lombok shares a similar cultural heritage with Bali and is linguistically related to the Balinese; however, Islam is the main religion on Lombok [22,23]. The population of Lombok consists mainly of two community types: the Sasakese, which is the indigenous population of Lombok, and the Balinese. There are also smaller numbers of Chinese, Javanese, Timorese (Flores) and expatriates. On Lombok, high numbers of free-roaming dogs are observed. These free-roaming dogs seem to consist of ownerless dogs and poorly supervised owned dogs; however, it can be difficult to distinguish the two groups. There is no reliable dog registration data available on Lombok, despite efforts made by the Livestock and Animal Health Agency at provincial and district levels in 2010 and 2011 to register owned dogs.

The study sites purposely selected for this survey were one city and one district on Lombok, representing an urban area and a rural area, considered high risk for the entry of rabies and inhabited by more than one ethnic group. Mataram City, the selected urban area, is located near the west coast of Lombok and is the major commercial center on the island (and the capital of NTB province). This site was selected as it includes all ethnic communities living on Lombok Island, has a higher socio-economic status than other areas of Lombok and a high density of people and dogs. The selected rural area was West Lombok District due to its close proximity to rabies-infected Bali, the presence of several informal ports, and the presence of more than one ethnic group.

Within the two sites, we purposively selected: Cakranegara District (urban) and Batu Putih village (rural). Cakranegara District, a central business area in Mataram City, is inhabited predominantly by Balinese and Sasakese ethnic groups with a minority of other ethnic groups, and has a high density of people and dogs compared to other districts. Batu Putih village was selected because it has the informal port which is closest to Bali of all the ports in Lombok (45 minutes by boat to the south-eastern coast of Bali). This informal port has no quarantine post and is often visited by boats from Bali. Furthermore, this village is inhabited predominantly by Balinese and Sasakese ethnic groups and fishing is an important occupation.

### Dog management practices survey

The study population for this survey was dog-owning households at the urban and rural sites. These were divided into two categories according to the ethnic group: Balinese and non-Balinese. The required sample size to estimate the proportion of households owning a pedigree dog in each ethnic group was calculated (Win Episcope 2.0, http://www.clive.ed.ac.uk) assuming a 95% confidence level, 5% precision and an assumed pedigree ownership proportion of 20%. For the urban site an infinite population was assumed (>1,000 households) whilst a finite population of 150 households per ethnic group was assumed in the rural site. The target sample size per ethnic group was 235 and 94 households in the urban and rural site, respectively.

Within the Cakranegara district, 12 of 51 Lingkungan (a term for a village in an urban setting) were selected using the random process described below for this study. At each of these 12 locations, the interview team consulted with the community leader (Kepala Lingkungan) to seek information on dog-owning households in the area. Of the 12 locations, five were known to be inhabited by mainly people of the Balinese ethnic group. At each of these five locations, 50 Balinese dog-owning households were conveniently selected and interviewed. Households were approached by an interview team in conjunction with the community leader; if the dog owner or the person who took care of the dog was present, he/she was interviewed.

In contrast, at the remaining seven locations, where most people were from a non-Balinese (Sasakese, Chinese and other) ethnic group, the community leader did not have information on the dog-owning households residing at these locations. Thus an alternative convenience approach was implemented after consultation with a veterinarian from Mataram City, who provided addresses of approximately 80 non-Balinese dog-owning households from across the city. Of these, 32 were identified and interviewed, who also provided information on 18 additional households. This resulted in 50 non-Balinese dog-owning households located in an urban area being interviewed. As a result the non-Balinese participants also included households in Mataram City outside Cakranegara district.

At the rural site, although the required sample size was 94 households for each ethnic group, only 60 Balinese dog-owning households were identified by the village leader. No information about dog ownership among the non-Balinese (Sasakese) was available and therefore the same number was targeted for this ethnic group. For the Balinese ethnic group, each household in the village was visited and an interview conducted when the occupant was at home at time of visit and if currently owning dog/s. A total of 50 interviews were completed. Contact and interviews with 50 Sasakese dog-owning households was achieved using the snowballing method with initial households identified by Balinese participants and the remainder by Sasakese participants.

### Questionnaire design and implementation

A structured questionnaire was designed to obtain data on number of dogs owned, dog function, dog management and dog bites from dog-owning households. The questionnaire was developed in English and translated into Bahasa Indonesian by an independent university academic, then back translated from Bahasa into English by a member of the Indonesian research team. Points of difference were identified and discussed by the two translators and clarified to determine the correct final wording in Bahasa. Minor modification of the questionnaire was performed after piloting it with ten dog-owning households at the urban and rural sites, including Balinese and non-Balinese households. The data obtained from the pilot trial were included in the data analysis. The questionnaire is available from the corresponding author upon request.

The questionnaire survey was conducted from January to April 2012 by the lead author and a team of eight people from the Research Centre for Rural Development, University of Mataram, NTB Province. This team had extensive experience in conducting questionnaire surveys, including animal health surveys in NTB province and had a 1-day training to become familiar with the dog-management questionnaire.

The questionnaire was administered via a face-to-face interview with the person responsible for the dog management at the household residence. The interviews were completed within 30 to 35 minutes and a rabies brochure and incentive of 20,000 IDR (about USD 2) were given to participants on completion of the interview. Ethics approval for this survey was obtained from the Ethics Committee at the University of Mataram, and participants provided written informed consent to participate in this study.

### Dog population estimation study

The aim of this counting exercise was to compare the size of the dog population in the urban and in the rural study areas. For the estimation of unowned dog population size, the same sites described above were used. At Cakranegara District, 12 of 51 Lingkungan were selected using the World Society for the Protection of Animals approach (http://www.animalsheltering.org/training-events/expo/expo-2013-handouts/hsi-humane-dog-management-gamboa1.pdf, accessed 20 May 2013) to achieve a random selection of Lingkungan for sampling that are well distributed across the chosen district, with each Lingkungan having a known and equal probability of selection. Due to logistic considerations, the total number of Lingkungan that could be managed by the research team was 12. Of these, seven were mainly inhabited by non-Balinese and five were mainly inhabited by Balinese. As results from the dog-owning household survey reported a low number of dog-owning households in the seven non-Balinese Lingkungan, only the five Lingkungan inhabited by the Balinese ethnic group were selected to conduct the unowned dog counting activity. At the rural site (Batu Putih village, West Lombok district), dog counting was conducted throughout the entire site.

A dog counting form was developed in English and translated into Bahasa Indonesia. Data recorded were village name, date and time of counting, weather at the time of counting, traffic condition (light or not) and visibility (good or not). The age, sex, body size and coat colour of each dog seen during counting were also recorded. Further the location where the dog was seen, the amount of garbage in the village (on a scale of 1 to 5) and the ‘sighting status’ (seen on day one, re-seen or not re-seen on day 2) were recorded.

The dog counting activity was conducted by the lead author and two personnel from the Animal Health Care Centre of the Agriculture, Marine and Fisheries Office in Mataram City. It was undertaken during April 2012, toward the end of the rainy season (November to May in Lombok). A preliminary visit was made to the urban and rural sites to find the most appropriate time of the day to conduct the unowned dog counting, according to the traffic and presence of street lighting. At Cakranegara District, the most feasible time was at night from 12:00 am to 02:00 am; whilst at Batu Putih village, the most feasible time was early morning between 6:00 am and 8:00 am, when traffic was minimal and natural light sufficient for observations (the lack of street lighting at this site prohibited dog counting at night-time). The counting was carried out at each location on two consecutive days.

The target dogs for the dog counting activity were unowned dogs defined as being free to roam and having no identifiable owner. As distinguishing free-roaming owned dogs from unowned dogs was challenging, dog owners (through village leaders during community gatherings) at these sites were asked to tether or collar their dogs during the dog counting activity.

The photographic recapture method was used to estimate the population of unowned dogs, implementing a modification of Schnabel’s variation of the Petersen-Lincoln Index using multiple recaptures [24]. As Beck stated, the individual differences between dogs make it possible to recognize individuals and to determine whether or not a dog had been previously photographed, i.e. “recaptured”. For this activity, a four cylinder motorbike run at a low speed was used along pre-defined routes to carry out the dog counting. At each site, the route comprised main roads and by-roads, which were all visited once. When an unowned dog was encountered, the observer photographed it, and recorded the location as well as the other information on the dog counting form. Places with potential for hiding, such as drains, under cars and behind garbage containers were also observed. This methodology has been successfully used recently in neighbouring Timor Leste [18].

Permission to undertake dog counting was obtained from community leaders with Cakranegara District and Batu Putih village. This field study did not involve endangered or protected species, and animal ethics committee approval was not required since no handling of dogs or activities that would change their behaviour were undertaken.

### Data management and analysis

Data were entered into Microsoft Excel and subsequently checked for missing and extreme values against the hardcopy questionnaires. Standard descriptive analyses were conducted using Genstat 14^th^ edition (PC/Windows, 2007, VSN International Ltd., Hemel Hempsted, UK). Categorical data were described using frequency tables and continuous variables using mean, median and range. Standard statistical tests were used to determine differences between categories for categorical variables (chi-square) and between means for continuous variables (t-test). The estimated number of unowned dogs was calculated using a Chapman estimate, with corresponding variance ([24]; Equation 2.1 and Equation 2.2) and 95% confidence interval.

## Results

### Dog ownership

Four hundred dog-owning households were interviewed. At the urban site, the households comprised 250 Balinese and 50 non-Balinese, including seven different ethnic groups: Chinese (n = 22; 44%), Sasakese (11, 22%), Javanese (8, 16%), Flores (6, 12%), Batak (1, 2%), Bima (1, 2%) and expatriate (1, 2%). At the rural site, there were 50 Balinese and 50 non-Balinese (all Sasakese) households.

Among the urban dog-owning households, 186 (62%) owned one dog, 81 (27%) owned two dogs, 22 (7.3%) owned three dogs and 11 (3.7%) owned between four and six dogs. Of the 100 rural dog-owning households, 57 (57%) owned one dog, 31 (31%) owned two dogs, three (3%) owned three dogs and nine (9%) owned between four and seven dogs. The mean number of dogs owned per household at the urban (mean 1.6) and rural sites (mean 1.7) was not significantly different (P = 0.938).

### Type of dog kept

The most common type of dog kept by all ethnic groups interviewed was non-pedigree, except for the Chinese ethnic group, in which 77.3% of households owned pedigree dogs. The percentage of households owning pedigree dogs was higher at the urban site than the rural site (Table 1).

**Table 1:** Type of dog owned by the 400 dog-owning households interviewed at two sites on Lombok, Indonesia, in 2012.

Site / Ethnic group	Dog type owned		
	Local	Pedigree	Local and pedigree
*Urban*			
Balinese (n = 250)	74% (185/250)	18.8% (47/250)	7.2% (18/250)
Non-Balinese (n = 50)	50% (25/50)	48% (24/50)	2% (1/50)
Chinese (n = 22)	18.2% (4/22)	77.3% (17/22)	4.5% (1/22)
Sasakese (n = 11)	72.7% (8/11)	27.3% (3/11)	0
Javanese (n = 8)	75% (6/8)	25% (2/8)	0
Timorese (Flores) (n = 6)	83.3% (5/6)	16.7% (1/6)	0
Other^a^ (n = 3)	66.7% (2/3)	33.3% (1/3)	0
*Rural*			
Balinese (n = 50)	96% (48/50)	2% (1/50)	2% (1/50)
Non-Balinese^b^ (n = 50)	98% (49/50)	2% (1/50)	0

^a^ Batakese ethnic group, Bimanese ethnic group and expatriate

^b^ Sasakese

### Dog function

Guard dog was the main function of dogs kept by both ethnic groups (Balinese and non-Balinese) at both sites: guarding the house at the urban site and guard the house and plantation at the rural site. Dogs were kept solely as pets by Balinese, Chinese, Javanese, Timorese and expatriate households at the urban site, with the proportion notably higher for Chinese households. Only one Chinese household reported keeping dogs to trade (Table 2).

**Table 2:** Function of dog reported by the 400 dog-owning households interviewed at two sites on Lombok, Indonesia, in 2012.

Site / Ethnic group	Dog function			
	Guard dog	Pet dog	Pet and guard dog	Trading purpose
*Urban*				
Balinese (n = 250)	61.6% (154/250)	8.4% (21/250)	30% (75/250)	0
Non-Balinese (n = 50)	48% (24/50)	24% (12/50)	26% (13/50)	2% (1/50)
Chinese (n = 22)	27.3% (6/22)	36.4% (8/22)	31.8% (7/22)	4.5% (1/22)
Sasakese (n = 11)	90.9% (10/11)	0	9.1% (1/22)	0
Javanese (n = 8)	37.5% (3/8)	25% (2/8)	37.5% (3/8)	0
Timorese (Flores) (n = 6)	66.6% (4/6)	16.7% (1/6)	16.7% (1/6)	0
Other^a^ (n = 3)	33.3% (1/3)	33.3% (1/3)	33.3% (1/3)	0
*Rural*				
Balinese (n = 50)	86% (43/50)	0	14% (7/50)	0
Sasakese (n = 50)	100% (50/50)	0	0	0

^a^ Batakese ethnic group, Bimanese ethnic group and expatriate

### Dog demographics and confinement

**Fig 1:**
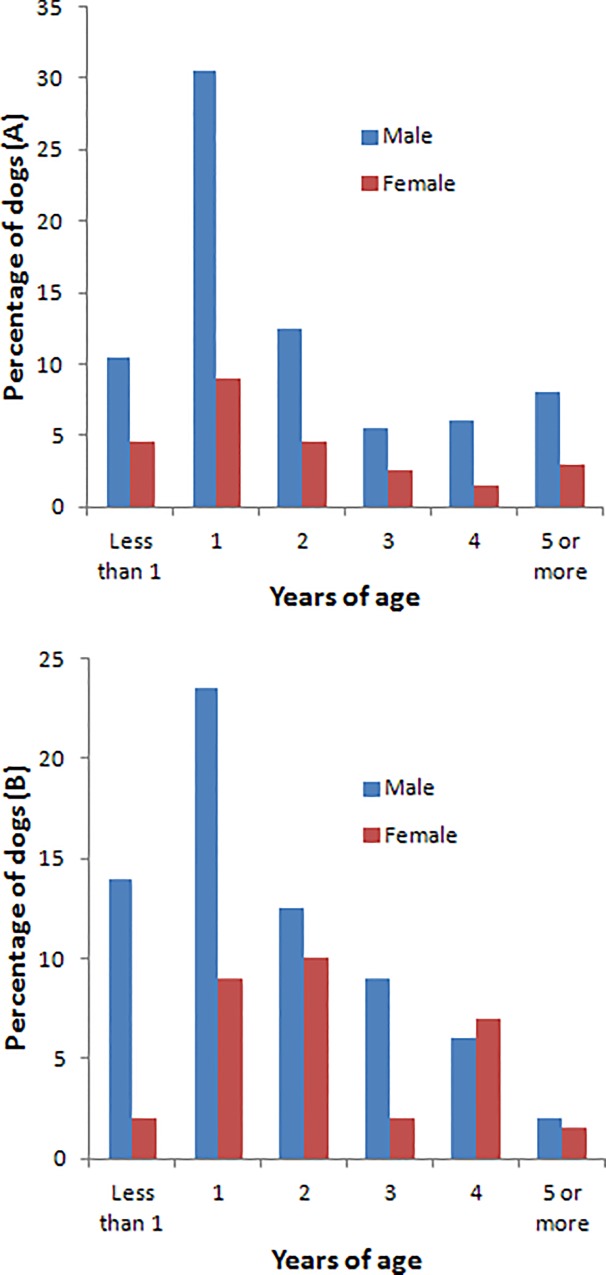
Age and sex distribution of 638 dogs (468 urban, 170 rural) owned by households interviewed at urban (A) and rural (B) sites on Lombok, Indonesia, in 2012.

A total of 638 dogs were kept by the households surveyed. Male dogs were more numerous at both study sites (P = 0.104), constituting 74.1% of dogs at the urban site (*n* = 468) and 67.6% at the rural site (*n* = 170) (Fig 1), with a male:female ratio of 1:0.3 at the urban site and 1:0.5 at the rural site. The same median age was seen for dogs living at the rural and urban sites (Table 3). The majority of dogs owned at both sites were non-pedigree or local breed, with the highest percentage of non-pedigree dogs at the rural site (Table 3).

**Table 3:** Demographics and confinement of 638 dogs (468 urban, 170 rural) owned by households interviewed at urban and rural sites on Lombok, Indonesia, in 2012.

	Urban site	Rural site
*Demographics*		
Age in years mean (median, range)	2.9 (2, 0.08–15)	2.1 (2, 0.08–10)
Sex		
Male	74.1% (347/468)	67.6% (115/170)
Female	25.9% (121/468)	32.4% (55/170)
Dog type		
Pedigree	29.7% (139/468)	1.8% (3/170)
Non-pedigree	70.3% (329/468)	98.2% (167/170)
*Confinement*		
Totally confined	29.3% (137/468)	0
Semi-free roaming	25.6% (120/468)	29.4% (50/170)
None (always allowed to roam)	45.1% (211/468)	70.6% (120/170)

The movement of dogs living at the urban site was better controlled than at the rural site. Of the 468 dogs at the urban site, 137/468 (29.3%) were restricted at all times, whereas dogs at the rural site were either always allowed to roam outdoors or semi-free roaming (roaming freely for some hours each day), and none of the dogs were restricted at all times (Table 3). The distribution of dogs by confinement categories was significantly difference between urban and rural sites (P <0.001). At the urban site, more pedigree dogs (59.9%; 82/139) than non-pedigree dogs (16.7%; 55/329) were confined (P <0.001) (Table 4).

**Table 4:** Management of 635 dogs owned by 400 interviewed households in urban and rural sites on Lombok, Indonesia, in 2012.

Site / Ethnic group	Dog Type	Number of dogs	Confined	Semi free roaming	None (always allowed to roam)
*Urban*					
Balinese household	Pedigree dog	103	51.5% (53/103)	14.6% (15/103)	33.9% (35/103)
	Local breed dog	296	16.9% (50/296)	29.4% (87/296)	53.7% (159/296)
Non-Balinese household					
Chinese	Pedigree dog	26	92.3% (24/26)	7.7% (2/26)	0
	Local breed dog	6	66.7% (4/6)	33.3% (2/6)	0
Sasakese	Pedigree dog	3	66.7% (2/3)	33.3% (1/3)	0
	Local breed dog	9	0	0	100% (9/9)
Javanese	Pedigree dog	4	50% (2/4)	50% (2/4)	0
	Local breed dog	6	16.7% (1/6)	66.6% (4/6)	16.7% (1/6)
Flores	Pedigree dog	1	100% (1/1)	0	0
	Local breed dog	8	0	75% (6/8)	25% (2/8)
Other ethnic	Pedigree dog	2	0	0	100% (2/2)
	Local breed dog	4	0	25% (1/4)	75% (3/4)
Total	Pedigree dog	139	59.9% (82/139)	14.4% (20/139)	26.6% (37/139)
	Local breed dog	329	16.7% (55/329)	30.4% (100/329)	52.9% (174/329)
*Rural*					
Balinese household	Pedigree dog	2	0	0	100% (2/2)
	Local breed dog	73	0	42.5% (31/37)	57.5% (42/73)
Sasakese household	Pedigree dog	1	0	100% (1/1)	0
	Local breed dog	94	0	19.1% (18/94)	80.9% (76/94)
Total	Pedigree dog	3	0	33.3% (1/3)	66.7% (2/3)
	Local breed dog	167	0	29.3% (49/167)	70.7% (118/167)

### Dog bite cases

A total of 27 households that were interviewed reported 29 dog bite cases during the last five years. One Balinese household reported 3 dog bite cases. At the urban site, 12 Balinese households reported 14 dog bite cases: the median age of the 14 people bitten was 34 (1−77) years. One non-Balinese (Timorese-Flores) household reported one dog bite case, aged 17 years old. At the rural site, 6 dog bite cases were reported by 6 Balinese households, median age 14 (5−65) years, while 8 non-Balinese households reported 8 dog bite cases, median age 37.5 (10−65) years.

Of the total 29 dog bite cases, half were children and young adults (5 aged 1−5 years, 2 aged 6−10, 5 aged 11−15, 3 aged 16−20). There were more dog bite cases in males (62.1%, 18/29) than in females (37.9%, 11/29) (P = 0.194). The leg was the part of the body reportedly bitten the most (68.9%), then hand/arm (17.4%), stomach (6.9%), hip (3.4%) and shoulder/neck (3.4%). The dog bite cases were frequently bitten by another person’s dog or their own dog (48.3% and 44.8%, respectively); only 6.9% of bite cases were bitten by a free-roaming dog. Only five people at the urban site and one person at the rural site went to hospital for treatment of bite wounds: all belonged to the Balinese ethnic group. The other cases treated their wound/s at home by cleaning the wound with or without application of iodine. Other types of treatment, such as using traditional medication, were reported at both the urban and rural sites, but were more common at the rural site. Non-treatment of the wound was higher at the rural site (Table 5).

**Table 5:** Treatment applied to the dog bite wound of the 29 dog bite cases recorded during a survey of dog owning households on Lombok, Indonesia, in 2012.

Site / ethnic group	Seek treatment at hospital	Clean the wound and apply iodine	Only clean the wound	Traditional medication	No treatment at all
*Urban*					
Balinese (n = 14)	35.7% (5/14)	35.7% (5/14)	0	21.4% (3/14)	7.1% (1/14)
Non-Balinese (n = 1)	0	100% (1/1)	0	0	0
*Rural*					
Balinese (n = 6)	16.7% (1/6)	16.7% (1/14)	16.7% (1/6)	33.2% (2/6)	16.7% (1/6)
Non-Balinese (n = 8)	0	0	0	62.5% (5/8)	37.5%(3/8)

### Estimation of unowned dog population

During dog counting, it was assumed that all dogs present without a collar on the counting days were ownerless dogs because dog owners had been asked to confine or collar their dogs. Only one dog with a collar was viewed during that time. The majority of houses at the two sites were fenced. Only dogs present outside residences' fences were counted. Unowned dogs were seen near garbage piles, on the streets, and sleeping in front of closed shops, around unfinished buildings and in empty water drainage. The estimated number of unowned dogs at the urban site was 180 ± 14.2 SD and at the rural site 65 ± 7.6 SD (Table 6).

**Table 6:** Demographics of unowned dogs (158 urban, 58 rural) observed during counting at urban and rural sites in Lombok, Indonesia, in 2012.

	Urban site	Rural site
Demographics		
Age^a^		
Puppy	7.6% (12/158)	24.1% (14/58)
Young	8.2% (13/158)	8.6% (5/58)
Adult	84.2% (133/158)	67.2% (39/58)
Sex		
Male	81.6% (129/158)	69% (40/58)
Female	11.4% (18/158)	10.3% (6/58)
Sex not seen	7% (11/158)	20.7% (12/58)
Seen on both days	47.5% (75/158)	46.6% (27/58)

^a^ Ages of the dogs were distinguished by the body size.

## Discussion

Of the dog-owning households interviewed in this survey, the majority (at both urban and rural sites) owned only one dog, most commonly non-pedigree and more commonly male. Dogs are used to guard houses, and in rural areas to guard plantations against monkeys and feral pigs destroying valuable crops. Based on common local understanding, female puppies are likely to be abandoned soon after birth (drh. Aminurrahman *pers*. *comm*., 2013). The preference appears to be related to guarding duties, given the majority of interviewed households keep dogs for this reason. A preference for keeping dogs for guarding purposes has also been observed in other countries such as Madagascar, Tanzania and the Philippines [25–27].

We found that among the Chinese households interviewed, most owned pedigree dogs rather than local non-pedigree dogs. The other ethnic groups were different, with the percentage of households owning non-pedigree dogs being higher than those owning pedigree dogs. However, there appears to be a growing trend among ethnic groups living in urban settings on Lombok to own pedigree dogs. Unlike Bali with its native dog breed (Kintamani) and a variety of dog breeds sold by pet shops, traditional live animal markets, dog traders and dog breeders; on Lombok Island, pedigree dogs are not easily found. This creates the possibility of further illegal dog imports not only for household purposes, but also for trading purposes.

In this survey, most (71%) owned dogs were semi-free roaming or free-roaming at the urban site, and all owned dogs at the rural site were semi-free roaming or free-roaming. A substantial number of roaming dogs is a threat for rabies transmission, should an incursion occur in Lombok, and for transmission of other zoonotic diseases. Therefore, control and management of owned dogs is needed through education of dog owners by relevant government authorities and veterinarians.

This survey recorded dog bite cases at both urban and rural sites. The reported dog bite cases were common in children/young adults (up to 20 years old) and more common in males than females. This distribution has also been found in other countries in Asia, such as Bangladesh, Bhutan and India.[28−30] Human males and individuals below <20 years of age are known risk factors for rabies incidence related to dog bites [30–33]. In this survey the leg was the body part most frequently bitten, as reported for dog bites in Bhutan [30]. Appropriate wound treatment − such as washing the wound with soap, application of antiseptic after washing, and going to the hospital − was reported by only a small number of households (more often Balinese households at both urban and rural sites). For the remaining bite cases, treatment was traditional medication or none at all. The negligent care of dog bites found in this survey is similar to that found in Bangladesh [29]. Proper wound care is part of post-exposure prophylaxis for rabies; thus, information on wound care should be made available in Lombok as part of a rabies prevention strategy. A reporting system also needs to be established at the village level, which would allow surveillance of dog bite incidence to support detection of a rabies incursion.

The survey conducted to estimate dog population size used the WSPA approach to select locations at the urban site. Of the 12 locations selected, only five Lingkungan that were inhabited mainly by the Balinese ethnic group were actually used. Given that dogs are more tolerated and appreciated in these communities, targeting Balinese locations provides an estimate of the likely maximum dog population size to inform rabies exposure assessment and response strategies. According to our survey, the estimated number of unowned dogs at the urban site is higher than at the rural site (180 dogs versus 65 dogs, respectively). It appears that the type of garbage, such as fish waste, leftover meals and other household kitchen garbage seen at the urban site provides food for the unowned dogs. A study conducted at Sao Paulo also showed the importance of garbage for free-roaming dogs [34].

Lack of dog population control can lead to higher numbers of free-roaming dogs [35]. Approaches to population control of free roaming dogs are generally based on culling and sterilisation programs [21,36]. On Lombok island, attempts to reduce the free-roaming dog population have been implemented through a dog culling program (two to three times a year) and intermittent sterilisation programs (however, this costly exercise is limited by budgetary constraints). The culling program is limited to unowned dogs while the sterilisation program is for both catchable unowned dogs and owned dogs, with permission from the owners. These programs started in 2009, after rabies spread on neighbouring Bali in late 2008. However, there has been no evaluation of the impact of these programs on the free-roaming dog population on Lombok. Also, the sterilised dogs are not identified (e.g. collar or tag) and the culling program is not targeted (drh. Aminurrahman personal communication, 2011); thus, it is possible that the sterilised dogs may be culled as well. Capturing ownerless and unwanted owned dogs and housing them in animal shelters, as occurs in Ireland, UK, Australia and New Zealand [37–39] seems far beyond the financial capacity of Lombok as an island in a developing country.

Feasible methods of unowned dog population reduction on Lombok may include reducing dog access to garbage disposal areas: for example, fencing the dump area. Further, regular sterilisation of free-roaming dogs (unowned and owned dogs) will also assist population reduction. Inexpensive identification should be provided for the sterilised dogs. Education of dog owners to promote responsible dog ownership is also necessary, as many semi-free roaming and free-roaming owned dogs are seen on Lombok. Owned dogs that are allowed to roam freely will likely contribute to the unowned dog population (through breeding) and to disease transmission.

Dog counting at the urban and rural sites provided initial data about the unowned dog population in urban and rural Lombok. The main limitation of this was utilisation of a simple dog population estimation method to estimate the unowned dog population. This method is not the best way to estimate the number of dogs [40]. Other methods, such as the Bayesian method used in Chad [41] may improve the accuracy of survey results. However, the simple method used in this survey was adequate to provide an initial estimate of the ownerless dog population at urban and rural sites on Lombok, and was appropriate given the limited time period for dog counting (two days at each site) that could be completed. In addition, the interview survey conducted should be repeated to validate its findings and also to monitor changes in dog management practices.

Promoting responsible dog ownership (dog registration and identification, vaccination, neutering and confinement) on Lombok should is an important component of activities to prevent the introduction and establishment of rabies on Lombok. However, this needs to consider the diversity of cultural and religious beliefs. Dog management could be included in community-based programs on rabies and thus presented in the context of rabies prevention, surveillance and early detection of a rabies incursion.

## Conclusion

The pattern of dog ownership on Lombok varies between ethnic groups. Education of dog owners about the control and management of their dogs is needed as part of a program to reduce the likelihood of a potential rabies incursion becoming established. There needs to be an emphasis on responsible dog ownership (particularly dog confinement and desexing).

## Supporting Information

S1 Dataset Dataset. (XLSX)
